# Role of Non-Coding RNAs in White and Brown Adipose Tissue Differentiation and Development

**DOI:** 10.3390/ncrna11030030

**Published:** 2025-04-29

**Authors:** Lea Sleiman, Sorina Dinescu

**Affiliations:** 1 Department of Biochemistry and Molecular Biology, University of Bucharest, 050095 Bucharest, Romania; sleiman.lea@s.bio.unibuc.ro; 2 Research Institute of the University of Bucharest (ICUB), 050663 Bucharest, Romania

**Keywords:** miRNA, WAT, BAT, lncRNA, circRNA, hADSCs, adipogenic differentiation

## Abstract

Adipocyte differentiation is a complex process in which pluripotent mesenchymal stem cells (MSCs) differentiate and develop into mature fat cells, also known as adipocytes. This process is controlled by various transcription factors, hormones, and signaling molecules that regulate the development of these cells. Recently, an increasing number of non-coding RNAs (ncRNAs), especially microRNAs (miRNAs), have been established to be involved in the regulation of many biological processes, including adipocyte differentiation, development, metabolism, and energy homeostasis of white and brown adipose tissue. Several in vitro and in vivo studies reported the significant role of ncRNAs in either promoting or inhibiting adipocyte differentiation into white or brown fat cells by targeting specific transcription factors and regulating the expression of key adipogenic genes. Identifying the function of ncRNAs and their subsequent targets contributes to our understanding of how these molecules can be used as potential biomarkers and tools for therapies against obesity, diabetes, and other diseases related to obesity. This could also contribute to advancements in tissue-engineering based treatments. In this review, we intended to present an up-to-date comprehensive literature overview of the role of ncRNAs, including miRNAs, long non-coding RNAs (lncRNAs), and circular RNAs (circRNAs), focusing particularly on miRNAs, in regulating the differentiation and development of cells into white and brown adipose tissue. In addition, we further discuss the potential use of these molecules as biomarkers for the development of novel therapeutic strategies for future personalized treatment options for patients.

## 1. Introduction

Adipose tissue, one of the largest organs of the body, is considered a highly complex and dynamic endocrine organ capable of responding to various environmental stimuli by secreting numerous growth factors, matrix proteins, enzymes, and hormones [1]. It plays a significant role in energy balance and fatty acid homeostasis. Adipose tissue depots are mainly found near organs. Cells within these depots are responsible for different functions, for instance, coordination of lipid storage by either increasing the number of adipocytes, a process known as hyperplasia, or by initiating the oxidation of stored energy [2]. In addition to its main role as a primary metabolic passive organ for energy storage, adipose tissue is also responsible for synthesizing and secreting various molecules known as adipokines. Adipokines, such as leptin, resistin, adipsin, adiponectin, visfatin, omentin, tumor necrosis factor alpha (TNFα), interleukin-6 (IL-6), monocyte chemoattractant protein-1 (MCP-1), and retinol-binding protein 4 (RBP4), are bioactive cytokines that regulate several physiological processes, including control of insulin sensitivity, metabolism, nutritional intake, and inflammation [3]. Adipocytes represent the main adipose tissue cell type and are mainly classified into white and brown adipocytes. These cells show characteristic morphological differences. Typically, white adipocytes present a single lipid droplet in their cytoplasm, whereas brown cells show multiple lipid droplets with an abundance of cytoplasmic mitochondrial organelles [4]. Adipocytes are known for their role in (i) energy storage in the form of triglycerides, (ii) regulation of the blood glucose level, (iii) production and release of adipocyte-specific proteins, and (iv) secretion of various effector molecules such as exosomes, peptide hormones, inflammatory cytokines, and miRNAs [5]. Additionally, adipocytes within adipose tissue have been shown to modulate organ remodeling and regeneration [6,7,8]. Apart from adipocytes, the adipose tissue is composed of other types of cells that function and intercommunicate as a single unit, including endothelial cells, stromovascular cells, lymphocytes, immune cells, macrophages, fibroblasts, and stem cells, such as adipose-derived stem cells (ADSCs). The communication between these cells and the balance in their expression profile significantly favors the maintenance of energy homeostasis. Over time, numerous major regulators of white adipose tissue (WAT) and brown adipose tissue (BAT) have been identified, including transcription factors, epigenetic factors, metabolic components, and ncRNA molecules. Recently, several studies highlighted the significant role of miRNAs and other ncRNA molecules, including lncRNAs and circRNA, in regulating the differentiation process and function in both WAT and BAT. Therefore, in this review, we seek to summarize the work undertaken on ncRNA molecules, focusing on miRNAs, in regulating white and brown adipose tissue differentiation, by pointing out the function of individual molecules and their specific gene targets. The potential role of these molecules as biomarkers and therapeutic targets will also be briefly discussed.

## 2. White vs. Brown Adipose Tissue

Two types of adipose tissue exist: WAT and BAT. Cells isolated from both types present different characteristics and exhibit different morphological aspects, which give rise to specialized tissue properties and functions.

### 2.1. White Adipose Tissue (WAT)

**Figure 1:**
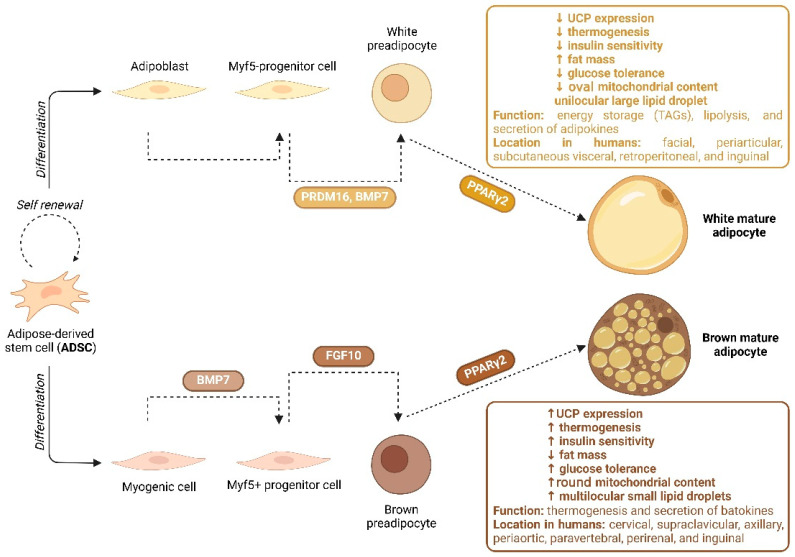
Biogenesis of white vs. brown adipocyte cells and their characteristics (created in BioRender.com, accessed on 20 February 2025). Abbreviations: MYF5−/+: myogenic factor 5−/+; PRDM16: positive regulatory domain zinc finger protein 16; BMP7: bone morphogenetic protein 7; PPARγ2: peroxisome proliferator-activated receptor gamma 2; UCP: uncoupling protein; TAGs: triacylglycerols; FGF10: fibroblast growth factor 10.

White adipose tissue (WAT) is found in the subcutaneous and visceral regions of the adipose tissue. The main function of this tissue is to store excess energy in the form of triglycerides [9]. It is also responsible for the secretion of adipokines [10], endocrine communication [11], and insulin sensitivity [12]. White adipocytes exhibit a spherical phenotype with a unilocular lipid droplet in the cytoplasm (Figure 1). Other cell types, such as preadipocytes, ADSCs, endothelial cells, fibroblasts, and immune cells, are also present in WAT, but in smaller numbers compared to white adipocytes [13]. Interestingly, studies have shown that hyperplasia of WAT is responsible for obesity and can cause insulin resistance due to the dysfunction of metabolic pathways [14,15,16].

### 2.2. Brown Adipose Tissue (BAT)

Brown adipose tissue (BAT) can be found mainly in the neck, mediastinum, and interscapular regions of newborns and is less abundant compared to WAT. Cells isolated from BAT typically present characteristics different from those isolated from WAT, being more prone to undergo the process of skeletal myogenic differentiation [17]. Brown adipocytes present a multilocular morphology with small lipid droplets dispersed throughout the cytoplasm. BAT is rich in iron-containing mitochondria and is regulated by the adrenergic signaling pathway responsible for its main function, which is thermogenesis [18] (Figure 1). The thermogenesis process is mainly controlled by the expression of a specific BAT marker, uncoupling protein 1 (UCP1), in the inner mitochondrial membrane. UCP1, a proton transporter, breaks down adenosine triphosphate (ATP) and oxidative respiration by disrupting the ATP-producing proton gradient, enabling energy expenditure in the form of heat as protons flow back into the mitochondrial matrix [19].

## 3. Stem Cells and Adipose Tissue Plasticity

### 3.1. Adipose-Derived Stem Cells (ADSCs)

Adipose-derived stem cells (ADSCs) represent a subset of MSCs characterized by their capacity to expand in vitro and their potential to differentiate into multiple cell lineages, including the adipogenic lineage, making them of great interest for therapeutic applications and in the field of regenerative medicine [20]. Significantly, ADSCs are not considered to be a homogenous population, but rather a heterogenous one. These cells are characterized according to the International Society for Cellular Therapy (ISCT) and the International Federation for Adipose Therapeutics and Science (IFATS). ADSCs can be identified by being (i) plastic-adherent; (ii) immunosuppressive; (iii) positive for expression of CD235a, CD31, CD45, CD73, CD90, CD105 and negative for CD11b, CD14, CD19, CD34, CD45, CD79, CD117 surface markers; and (iv) capable of differentiating into chondrocytes, osteoblasts, and preadipocytes [21,22]. Unlike MSCs isolated from bone marrow, ADSCs can be easily isolated in high numbers from adipose tissue using minimally invasive procedures such as liposuction. The subcutaneous tissues of the abdomen, arm, and thigh represent the main sources from which these cells can be obtained [23].

### 3.2. Adipogenic Differentiation Potential

Adipogenesis is defined as the process through which MSCs differentiate into adipocytes by committing to the adipose lineage. The process by which preadipocytes transform into mature adipocytes is characterized by several morphological and structural changes, such as a decrease in proliferation capacity and accumulation of lipid droplets. Numerous studies reported the expanded differentiation potential of ADSCs into multiple cellular lineages both in vitro and in vivo, including the chondrogenic, osteogenic, myogenic, neurogenic, and adipogenic lineages due to their mesodermal origin [20,24,25,26,27,28]. The adipogenesis of white and brown cells is controlled by hundreds of regulatory elements, including cellular signaling pathways, cytoskeletal and extracellular matrix (ECM) proteins, transcription factors, ncRNA molecules (such as miRNAs, lncRNAs, and circRNAs), and endocrine hormones. Non-coding RNAs are, in turn, regulated by a variety of upstream intrinsic and extrinsic factors during adipogenesis. These include (i) transcription factors [29,30]; (ii) hormones and endocrine signals that influence the expression of ncRNA molecules involved in different metabolic processes [31,32]; (iii) intracellular signaling pathways, such as the WNT/β-catenin pathway, transforming growth factor-beta/SMAD (TGF-β/SMAD) pathway, mitogen-activated protein kinase/extracellular signal-regulated kinases (MAPK/ERK) pathway, and phosphoinositide 3-kinase/protein kinase B (PI3K/AKT) pathway, which are involved in the activation or inhibition of transcription factors and ncRNA biogenesis and function [33,34]; (iv) epigenetic modifications such as DNA methylation, histone modifications and chromatin remodeling [35,36]; and (v) environmental stimuli including cold exposure and oxidative stress [37,38,39,40].

In the following section, we will focus on the function of two key regulators, transcription factors and miRNAs, in modulating the adipogenic differentiation into white and brown adipose tissue.

## 4. Transcription Factors Involved in WAT and BAT Development

More than 20 transcription factors have been reported to influence the formation of white and brown fat. The activity of the majority of these factors is dependent on four regulators: peroxisome proliferator-activated receptor gamma (PPARγ), CCAAT-enhancer-binding proteins (C/EBPs), PPARγ coactivator 1 alpha (PGC1α), and positive regulatory domain zinc finger protein 16 (PRDM16).

PPARγ, also known as glitazone reverse insulin resistance receptor or NR1C3, belongs to the nuclear receptor superfamily of ligand-activated transcription factors [41]. The PPARγ gene is transcribed from different promoters, yielding the production of two major protein isoforms, PPARγ1 and PPARγ2. Both isoforms are predominantly expressed in adipocytes; however, PPARγ2 is considered the master regulator of adipogenesis [42]. Additionally, it has been demonstrated that PPARγ2 is not only directly involved in adipose tissue development and differentiation, but it also increases insulin sensitivity by favoring fatty acid deposition and storage, as well as adiponectin secretion from adipocytes, such as fibroblast growth factor 21 (FGF21) [43,44].

C/EBPs belong to a family of highly conserved basic-leucine zipper proteins [45]. The C/EBP family of transcription factors includes C/EBPα, C/EBPβ, C/EBPγ, C/EBPδ, C/EBPε, and C/EBPζ [46]. The first transcription factors found to be involved in the process of adipogenic differentiation were C/EBPα, C/EBPβ, and C/EBPγ. However, the stage at which each of these factors is expressed varies; for example, the expression of C/EBPβ is induced earlier in the differentiation process and is considered significant for the activation of C/EBPα and PPARγ, thus initiating the process of adipocyte development [47]. On the other hand, C/EBPα is expressed later in adipogenesis and is mainly found in mature adipocytes, where it plays a crucial role in regulating insulin-dependent glucose uptake and lipid metabolism [48]. Similar to C/EBPα, the expression of C/EBPγ is also induced later during adipogenic differentiation, as it has been suggested to have rather a modulatory function. In summary, C/EBPα is involved in the later stages of adipogenic differentiation, but unlike C/EBPα and C/EBPβ, it is not considered crucial for the initial commitment of cells to the adipogenic lineage [49].

PGC1α is a member of the transcription coactivators family that plays a role in regulating cellular energy metabolism [50]. Upon activation, PGC1α induces the expression of genes responsible for the initiation of mitochondrial biogenesis and oxidative phosphorylation, resulting in tissue-specific gene alterations. In contrast, a reduction in the expression of PGC1α is linked to metabolic disorders, inflammation, and disrupted redox control [51]. In the study conducted by Shen et al., the role of PGC1α in adipocyte browning and improving obesity-related metabolic dysfunction was investigated. It was reported that PGC1α determined the conversion of white adipocytes into brown-like cells by upregulating the expression of UCP1, FGF21, and phosphorylated AMP-activated protein kinase (pAMPK) signaling. Moreover, mice with an increased expression of PGC1α showed enhanced levels of Heme oxygenase-1 (HO-1) secretion, increased mitochondrial biogenesis, respiration, and improved oxygen consumption [52].

PRDM16 is a positive regulatory domain zinc finger protein and a key regulator of adipose tissue metabolism and browning of fat cells [53]. Studies have shown that PRDM16 is critical for the induction of expression of BAT-related genes, such as PGC1α, PGC1β, PPARγ, UCP1, and type 2 deiodinase (DIO2) [54]. Moreover, this protein can regulate the cell fate transition from myogenic factor 5+ (MYF5+) cells to brown pre-adipocytes by forming a transcriptional complex with C/EBPβ, hence activating the expression of PPARγ and thereby inducing thermogenesis [55]. PRDM16 was also reported to inhibit WAT-related gene expression at the messenger RNA (mRNA) level, such as phosphoserine aminotransferase 1 (PSAT1), serine peptidase inhibitor 3ak (Serpin3ak), and resistin [56].

## 5. miRNAs as Key Genetic Regulators

MicroRNAs, also known as miRNAs, represent a category of short, non-coding, single-stranded RNA molecules with an average length of 22 nucleotides [57]. These molecules play a significant role in regulating gene expression and survival. Typically, miRNAs bind to the 3′ untranslated region (3′UTR) of target mRNAs, leading to subsequent degradation and translational inhibition of those mRNA molecules [58]. However, under certain conditions, miRNAs can interact with other regions and enhance translation or regulate transcription [59]. Interestingly, one miRNA molecule can regulate the expression of many different genes, and conversely, the expression of a single target gene can be regulated by multiple miRNAs functioning together [60]. Several factors influence the interaction of miRNAs with their specific target genes, including their (i) subcellular localization, (ii) abundance, and (iii) level of affinity with target mRNAs [59]. Generally, miRNAs are transcribed into primary transcripts called pri-miRNAs from DNA sequences by a canonical pathway. These transcripts are transcribed from different parts of the genome, including the introns of non-coding genes or protein-coding genes, exons of non-coding genes, or the intergenic regions found within the genome [61]. Following transcription, pri-miRNAs are further processed into precursor miRNAs or pre-miRNAs and finally into mature miRNAs [59]. Over time, numerous studies have reported the role of miRNA molecules in regulating the differentiation, development, and function of various tissues, including adipose tissue. In the following section, we will discuss the role of several miRNAs in regulating the differentiation of white and brown adipose tissue by presenting different studies from the literature highlighting their function.

### 5.1. miRNAs Involved in the Differentiation and Function of WAT

**Figure 2:**
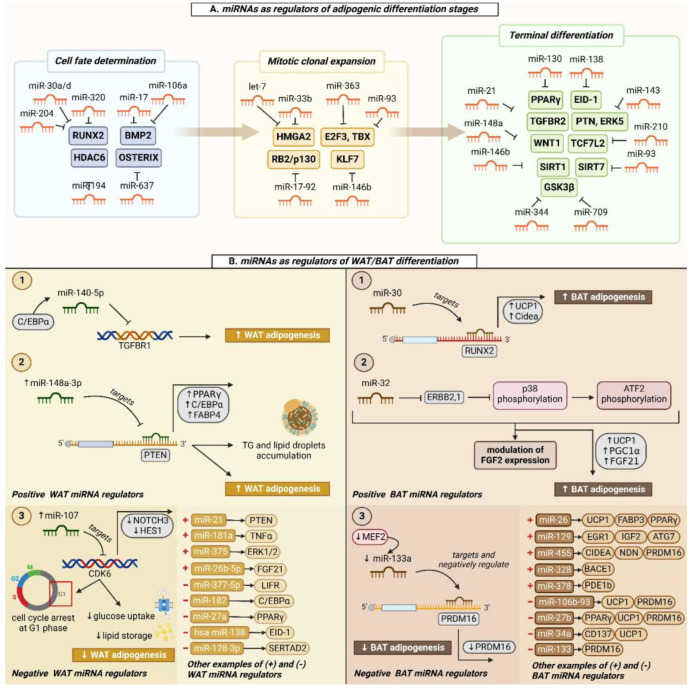
miRNAs and their subsequent targeting molecules in WAT/BAT adipogenesis. (**A**) miRNAs specific for regulating different differentiation stages of WAT. (**B**) Examples of miRNAs and their specific targets acting as positive or negative differentiation regulators of white and brown adipose tissue (created in BioRender.com, accessed on 19 February 2025). Abbreviation: RUNX2: runt-related transcription factor 2; HDAC6: histone deacetylase 6; HMGA2: high-mobility group AT-hook 2; E2F3: E2f transcription factor 3; TBX: T box 3; RB2/p130: retinoblastoma- related gene; KLF7: kruppel-like factor 7; PPARγ: peroxisome proliferator-activated receptor gamma; EID-1: EP300 interacting inhibitor of differentiation 1; TGFBR-: transforming growth factor beta-; PTN: pleiotrophin; ERK5: extracellular signal-regulated kinase 5; WNT1: wingless 1; TCF7L2: transcription factor 7 like 2; SIRT-: silent information regulator sirtuin-; GSK3β: glycogen synthase kinase 3 beta; C/EBPα: CCAAT-enhancer-binding protein alpha; WAT: white adipose tissue; BAT: brown adipose tissue; PTEN: phosphatase and tensin homolog; FABP-: fatty acid binding protein-; CDK6: cell division protein kinase 6; NOTCH3: neurogenic locus notch homolog protein 3; HES1: hairy enhancer of split 1; TNFα: tumor necrosis factor alpha; ERK1/2: extracellular signal-regulated kinases 1/2; FGF-: fibroblast growth factor-; LIFR: leukemia inhibitory factor receptor; SERTAD2: SERTA domain-containing 2; UCP1: uncoupling protein 1; ERBB2,1: Erb-B2 receptor tyrosine kinase 2 peptide 1; ATF2: activating transcription factor 2; PGC1α: PPARγ coactivator 1 alpha; MEF2: myocyte enhancer factor 2; PRDM16: positive regulatory domain zinc finger protein 16; EGR1: early growth response protein 1; IGF2: insulin-like growth factor 2; ATG7: autophagy-related 7; NDN: neurally differentiated embryonal carcinoma-derived protein; BACE1: beta-site amyloid precursor protein cleaving enzyme 1; PDE1b: phosphodiesterase 1b; CD137: cluster of differentiation 137.

The process of stem cell differentiation into mature white adipocytes involves a cascade of events, mainly divided into 4 stages: (i) Proliferation arrest: cells undergo growth arrest by contact inhibition indicating that confluence has been reached; (ii) Mitotic clonal expansion: preadipocytes undergo DNA replication and cellular duplication whereas committed preadipocytes receive a combination of adipogenic signals; (iii) Early differentiation: cell division is arrested and cells undergo phenotypic and cytoskeletal changes; also, lipid accumulation and induction of several adipogenic-related factors occurs, including C/EBPα, PPARγ2, and adipocyte determination- and differentiation-dependent factor 1/sterol regulatory element-binding protein 1c (ADD1/SREBP-1c); and finally, (iv) Terminal differentiation: cells become sensitive to insulin and several adipose-specific proteins are synthesized, such as fatty acid binding protein 4 (FABP4), perilipin, and fatty acid translocase/Cluster of differentiation 36 (FAT/CD36). Additionally, during this stage, there is an increase in the secretion levels of enzymes involved in triacylglycerol (TAG) metabolism [4]. Numerous miRNAs have been reported in the literature to play a role in regulating the different stages of adipogenic differentiation. These molecules mediate the process of WAT development and further differentiation. The function of different miRNAs and their stage of expression are listed in the table below (Table 1) and presented in Figure 2A.

Increasing data show that changes in miRNA expression profile play a significant role in regulating the differentiation of pre-adipocytes into mature white adipocytes. The expression of miR-21, miR-30, miR-103, miR148a, miR-181a, miR-210, miR320, miR-375, and miR-378 have been confirmed to promote adipogenesis [79,84,85], whereas the expression of miR-15a, miR-22, miR33b, miR-125a, miR-138, miR155, miR-205, miR-222, miR-224, miR-363, and miR448 has been shown to inhibit the differentiation process [69,86,87,88,89,90,91]. miRNAs regulating WAT adipogenesis can be grouped into positive WAT regulators and negative WAT regulators (Table 2).

#### 5.1.1. Positive WAT Regulators

Upregulation of miR-21 has been reported to significantly promote adipocyte differentiation and to increase both mRNA and protein expression profiles of adiponectin. miR-21 could also regulate adipocyte differentiation by decreasing the protein levels of activator protein-1 (AP-1) [97]. miR-26b is involved in adipogenesis by targeting the expression of the phosphatase and tensin homolog (PTEN) gene. In the study conducted by Song et al., silencing of miR-26b expression resulted in suppression of adipogenic differentiation and a decrease in lipid droplet and triglyceride synthesis and accumulation. Moreover, the expression of adipogenic-specific markers, including fatty acid-binding protein (AP2), C/EBPα, PPARγ2, and hormone-sensitive lipase (HSL), was also effectively downregulated upon loss-of-function of miR-26b [98]. miR-9-5p was reported to upregulate the expression of adipogenesis-specific genes, including PPARγ, Adipsin, and C/EBPα in Marrow Stromal Stem Cells (MSCs) by targeting the 3′UTR of wingless family member 3a (WNT3a) and inhibiting the WNT/β-catenin signaling. Compared to the control, MSCs overexpressing miR-9-5p showed increased lipid droplet deposition in the cytoplasm upon adipogenic differentiation [99]. miR-140-5p was identified as a positive regulator of differentiation into mature adipocytes. miR-140-5p directly targets the transforming growth factor beta 1 (TGFBR1) gene and is controlled transcriptionally by C/EBPα, which binds to and enhances the promoter activity of miR-140-5p (Figure 2B) [100]. miR-146a-5p was reported to promote adipogenic differentiation via the ERK1/2/PPARγ signaling pathway. In the study conducted by Wang et al., upregulation of miR-146a-5p was shown to increase intracellular lipid droplet accumulation and triglyceride levels. Additionally, the expression of PPARγ and FABP4 was observed to be significantly elevated at both gene and protein levels; however, the expression of miR-146a-5p target gene receptor protein-tyrosine kinase EEBB-4 (EEBB4) and ERK1/2 phosphorylation was decreased [101]. miR-146b was demonstrated to promote adipogenic differentiation by regulating the expression of silent information regulator sirtuin 1 (SIRT1), a protein involved in the maintenance of metabolic stability and promotion of fat deposition in WAT. miR-146b was shown to bind to the 3′UTR of SIRT1 and form a complex, hence regulating adipogenesis through increased acetylation of forkhead box O1 (FOXO1) [83]. miR-20a was reported to positively regulate adipogenic differentiation by directly targeting and inhibiting the activity of TGFBR2 and lysine-specific demethylase 6b (KDM6b) genes. Data from this study showed that the overexpression of miR-20a induced the activity of key adipocyte-mediated transcription factors C/EBPα, C/EBPβ, PPARγ, and AP2 [102]. miR-103 was identified as a positive regulator of adipogenesis. In the study conducted by Li et al., miR-103 was observed to function in promoting adipogenesis by blocking the activity of its target, myocyte enhancer factor 2D (MEF2D), a gene involved in regulating negatively key adipocyte factors. Overexpression of miR-103 increased the expression of adipogenic and lipid synthesis genes, including C/EBPα, PPARγ, FABP4, Fas cell surface death receptor (FAS), and acetyl-CoA carboxylase (ACC). Still, it blocked those of lipolysis genes, including hormone-sensitive lipase (Hsl) and adipose triglyceride lipase (ATGl). Additionally, miR-103 was proven to activate the protein kinase B/mammalian target of rapamycin (AKT/mTOR) signaling pathway by significantly increasing the phosphorylation of two key mTOR pathway genes, S6 kinase B1 (S6K1) and mTOR [103].

#### 5.1.2. Negative WAT Regulators

miR-143 was shown to regulate ADSCs’ differentiation by directly inhibiting a key member of the MAPK signaling pathway, MAP2K5. In the study conducted by Chen et al., expression of miR-143 was observed to be decreased following adipogenic induction; however, it increased after 3 days in culture, reaching its peak levels 7 days after induction. Interestingly, it was also demonstrated that this miRNA’s regulatory role depends on the differentiation stage. miR-143 overexpression during the mitotic clonal expansion stage inhibited the differentiation of ADSCs; on the contrary, overexpression of this miRNA molecule during the growth arrest stage or terminal differentiation stage promoted adipocyte differentiation [104]. miR-130 was shown to contribute to impaired adipogenic differentiation of MSCs during diet-induced obesity (DIO) by targeting and repressing the expression of adenomatosis polyposis coli downregulated 1 (APCDD1), which acts as an inhibitor of the WNT signaling pathway. miR-221 inhibited adipogenesis and attenuated the induction of master regulators PPARγ and C/EBPα. The expression of this miRNA was reported to be decreased in immortalized and primary human MSCs during adipogenesis, suggesting that the expression of miR-221 must be downregulated to ensure a normal differentiation process [87]. miR-107 has been demonstrated to have distinct regulatory roles in both pre-adipocytes and mature adipocytes. Ahonen et al. reported that the overexpression of miR-107 impairs differentiation and lipid accumulation via the downregulation of its target cell division protein kinase 6 (CDK6), yielding cell cycle arrest at the G1 phase of cell division. Additionally, neurogenic locus notch homolog protein 3 (NOTCH3), a signaling receptor involved in adipocyte differentiation, and its target hairy enhancer of split 1 (HES1), were also observed to be downregulated by miR-107. Reduced glucose uptake and triglyceride synthesis were observed in mature adipocytes, suggesting the dual role of miR-107 in inhibiting adipogenesis and lipid storage (Figure 2B) [105]. miR-128-3p was identified as a negative regulator of adipogenesis. Overexpression of miR-128-3p downregulated the expression of adipogenic-master marker genes, C/EBPα and PPARγ. Furthermore, the mRNA expression of FAS and AP2 was impaired during the process of adipogenic differentiation. miR-128-3p was also reported to obstruct lipid droplet accumulation and triglyceride levels by directly targeting and influencing the activity of SERTA domain-containing 2 (SERTAD2) gene, resulting in lipolysis and triglyceride hydrolysis [106].

### 5.2. miRNAs Involved in the Differentiation and Function of BAT

The main function of BAT, as mentioned earlier, is to maintain a constant body temperature, particularly in response to cold exposure, by dissipating energy in the form of heat. WAT and BAT share common pathways in adipogenesis, particularly during the differentiation and maturation of pre-adipocytes. Several signaling pathways involved in regulating the differentiation of WAT have been reported to be involved, as well as in the differentiation of BAT [107]. Changes in the expression of some miRNA molecules previously demonstrated as regulators of adipogenic differentiation in WAT were found to play a role in BAT regulation. These miRNA molecules target important genes involved in thermogenesis, leading to either the activation or inhibition of this process [108]. Similar to WAT, there exist two groups of miRNA molecules implicated in the process of brown tissue development. These molecules can either act as positive BAT regulators or negative BAT regulators (Table 3).

#### 5.2.1. Positive BAT Regulators

miR-182 and miR-203 were identified as regulators of brown fat tissue development. Knockdown of miR-182 and miR203 caused a significant decrease in the expression of specific BAT (UCP1, Cidea, PPARα, and PGC1α) and mitochondrial markers (Cyclooxygenase- (Cox7 and Cox8)) in primary adipocyte cells [121]. miR-30b/c overexpression induced the expression of thermogenic markers (UCP1 and Cidea); however, miR-30b/c knockdown determined the suppression of these markers’ expression. The authors of this study also reported an increase in mitochondrial respiration and thermogenic gene expression in primary adipocyte cells. Data from this study suggest the thermo-regulatory role of miR-30b/c in brown fat tissue development [122]. In an in vitro study conducted by Zaragosi et al., using human adipose-derived stem cells (hADSCs) as a model, the miR-30 family was shown to be a positive regulator of brown adipocyte differentiation, where the overexpression of miR-30a/d stimulated adipogenesis while its inhibition blocked this process. Additionally, the authors reported that the stimulation of adipogenesis induction by miR-30 was disrupted when the 3′UTR of transcription factor runt-related transcriptional factor 2 (RUNX2), a miR-30 target, was blocked (Figure 2B) [62]. miR-32 was identified as a BAT-specific positive regulator in mice. Inhibition of this miRNA activity in vivo determined a significant decrease in BAT thermogenesis, reduced cold tolerance, and browning of white fat tissue due to lowered FGF21 serum levels. Moreover, data from this study also suggested that miR-32 directly suppresses its target gene transducer of Erb-B2 receptor tyrosine kinase 2 peptide 1 (TOB1), and together can act as modulators of FGF21 expression and release from BAT by activating p38 mitogen-activated protein (MAP) kinase signaling (Figure 2B) [38]. In the study conducted by Sun et al., miR-193b-365 was reported to play a critical role in the proper development of brown fat cells by regulating the expression of key transcription factors and genes involved in thermogenesis, including UCP1 and PRDM16 [123]. miR-196a was reported to be essential for the generation of brown adipocyte-like cells in WAT in mice. It was shown that the upregulation of miR-196a suppresses the expression of its direct target, homeobox C8 (HOXC8), a gene known to be a repressor of brown adipogenesis. HOXC8 with histone deacetylase 3 (HDAC3), typically targets and inhibits the activity of C/EBPβ, a regulator of the brown fat gene program [124].

#### 5.2.2. Negative BAT Regulators

miR-133a has been reported to directly target the 3′UTR of PRDM16. Upon the commitment and differentiation of brown adipocytes, a significant decrease in the expression of miR-133 and upregulation of PRDM16 expression were observed. Additionally, a double knockout of two members of the miR-133 family, namely miR-133a1 and miR-133a2, resulted in an increase in brown and thermogenic gene programs in subcutaneous adipose tissue (Figure 2B) [122]. A similar result was observed in another study, where the downregulation of miR-133 levels in mice exposed to cold determined the upregulation of PRDM16 by targeting its 3′UTR region and the generation of brown adipocytes from adult skeletal muscle stem cells (satellite cells) [125]. miR-155 was shown to modulate the activity of key molecular regulators of BAT differentiation. Blocking the activity of miR-155 enhanced brown adipocyte differentiation and induced browning of white fat tissue, whereas the induction of miR-155 expression determined a reduction in brown adipose tissue development and function. Also, miR-155 and its target C/EBPβ were suggested to form a bistable mechanism that integrates hormonal signals to control both the proliferation and differentiation of adipocytes [126]. Another study showed that miR-155 in cooperation with nuclear factor-kappa B (NF-κB) forms a pro-inflammatory mechanism loop that could yield the amplification of an inflammatory state in adipocytes [127]. miR-34a has been demonstrated to modulate the browning of white fat tissue and energy metabolism by regulating the activity of fibronectin type III domain-containing protein 5 (FNDC5), a key protein involved in brown adipogenesis and thermoregulation [128].

## 6. Other Non-Coding RNA Molecules Involved in the Differentiation and Function of WAT and BAT

**Figure 3:**
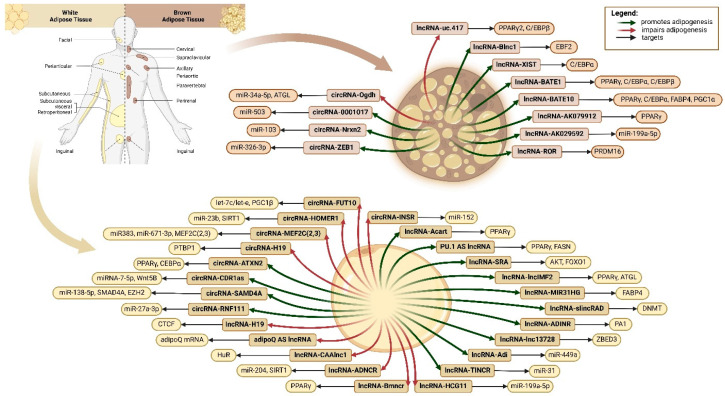
Schematic representation summarizing different lncRNA and circRNA molecules and their subsequent targets in white and brown adipocyte cells (created in BioRender.com, accessed on 20 February 2025). Abbreviations: PPARγ2: peroxisome proliferator-activated receptor gamma 2; C/EBP-: CCAAT-enhancer-binding protein-; EBF2: early B cell factor 2; FABP4: fatty acid binding protein 4; PGC1-: PPARγ coactivator 1-; PRDM16: positive regulatory domain zinc finger protein 16; ATGL: adipose triglyceride lipase; FASN: fatty acid synthase; AKT: protein kinase B; FOXO1: forkhead box O1; ATGL: adipose triglyceride lipase; DNMT: maintenance DNA methyltransferase 1; PA1: Pax2 transactivation domain interaction protein (PTIP)-associated protein 1; ZBED3: zinc finger BED domain-containing 3; SIRT1: silent information regulator sirtuin 1; HuR: Hu antigen R; CTCF: CCCTC-binding factor; SMAD4A: sterile alpha motif domain containing 4A; EZH2: enhancer of zeste homolog 2; WNT5B: wingless family member 5B; PTBP1: polypyrimidine tract-binding protein 1; MEF2C(2,3): myocyte enhancer factor 2C(2,3).

The following section of this review describes other members of the ncRNA family of molecules that have been reported in the literature to be responsible for the regulation of white and brown adipose tissue expansion and growth. Additional examples regarding the regulatory role, whether positive or negative, and the targets of these non-coding RNAs, are mentioned in Table 4 and presented in Figure 3.

### 6.1. Long Non-Coding RNAs (lncRNAs)

Long non-coding RNAs (lncRNA) represent a type of endogenous RNA transcript with a size that ranges from 200 nucleotide residues to 20 kb [146]. Earlier, lncRNAs were believed to have little or no functional importance and were considered transcriptional noise [147]. However, later studies indicated that these molecules play a significant role in a wide range of complex biological processes, and in some cases, they even encode for small peptides [148,149,150]. LncRNAs can interact with numerous molecules such as DNA, mRNAs, miRNAs, and other protein complexes and can influence the regulation of gene transcriptional activation, remodeling of chromatin, binding of miRNAs, and stabilization of proteins [151]. Regarding their cellular distribution, lncRNAs are mainly localized in the nucleus, but they can also be found in lower numbers in the cytoplasm [152]. In contrast to mRNA, lncRNAs are purely conserved among species with low expression rates, this expression being cell- and tissue-specific, depending on the localization of these molecules [153]. In recent years, with the help of advanced techniques such as high-throughput sequencing and microarray technology, numerous studies reported the function of lncRNAs as coregulators of adipogenesis and the development of white and brown adipose tissue [154,155,156,157]. Binding sites for the key transcription factor in adipocyte differentiation PPARγ are present in the promoter sites of many lncRNA genes [158]. Based on previous studies, a regulatory feedback network including lncRNAs, miRNAs, and mRNAs for the regulation of adipogenic differentiation of ADSCs has been proposed to validate the functions and mechanisms of lncRNAs in vitro. For instance, lncRNA-lnc13728 promoted hADSCs adipogenic differentiation by binding to zinc finger BED domain-containing 3 (ZBED3), a transcription factor implicated in the inhibition of the Wnt/β-catenin pathway. The expression of lncRNA-lnc13728 was significantly upregulated during hADSC differentiation, whereas its knockdown blocked the subsequent expression of adipogenesis-related genes at both mRNA and protein levels, in addition to decreasing lipid droplet synthesis and accumulation [159]. LncRNA-Adi was reported to promote the adipogenesis of ADSCs by interacting with miR-449a and together promoting the expression of CDK6 via activation of the retinoblastoma protein-E2F transcription factor 1 (pRb-E2F1) pathway. Similar to the previous study, adipogenic differentiation was impaired upon lncRNA-Adi knockdown [160]. LncRNA-TINCR was shown to modulate and enhance the adipogenic differentiation of hADSCs by negatively regulating the expression of miR-31. Interestingly, lncRNA-TINCR is activated upon the binding of C/EBPα to its promoter region [161]. LncRNA-HCG11 expression in vitro was decreased during the adipogenic differentiation of human adipose-derived mesenchymal stem cells (hADMSCs). LncRNA-HCG11 was also found to target miR-204-5p by binding to the 3′-UTR region of SIRT1, hence inhibiting adipogenic differentiation. During different differentiation stages, the expression of several adipogenic-specific markers, including C/EBPα, FABP4, PPARγ2, ACC, and FAS, was significantly decreased [162]. LncRNA-AK029592 has been identified to play a significant role in regulating the thermogenic program by promoting the browning of ADSCs. LncRNA-AK029592 was also determined to interact with miR-199a-5p to initiate the activation of the thermogenic gene and protein program [163]. LncRNA-ROR upregulation by adenovirus type 36 (Ad36) was reported to promote hADSCs’ differentiation into brown adipocytes. mRNA and protein expression levels of BAT-related markers (PRDM16, UCP1 CIDEA, FGF21, and DIO2), as well as mitochondrial respiratory chain- and phosphorylation-specific enzyme markers (ATP6, ATP50, NADH dehydrogenase 2 (ND2), and cytochrome C oxidase subunit 5B (COX5B)) were significantly increased during the browning of hADSCs [164]. Besides the role of lncRNAs in regulating the differentiation process of ADSCs into adipose tissue, some molecules were also reported, as mentioned earlier, as being specific for either white or brown adipose tissue expansion. LncRNAs implicated in this process of differentiation can be divided into positive and negative regulators of adipogenesis (Table 4).

### 6.2. Circular RNAs (circRNAs)

Circular RNAs (circRNAs) are defined as a class of covalently closed strands of ncRNAs which, unlike linear RNA, do not present in their structure a 5′-end cap or a 3′-end poly (A) tail [165]. Due to this unique feature, circRNAs are highly stable and resistant to exonuclease-mediated degradation [166]. Initially, circRNAs were considered to be the result of splicing errors. However, with the development and advancement of high-throughput sequencing technologies and bioinformatics techniques, hundreds of circRNAs were characterized in cells and tissues of different species and determined to be involved in numerous developmental and physiological conditions [167]. In the last few years, the molecular function of circRNAs in adipogenic differentiation has been extensively studied. Several circRNA molecules were shown to be implicated in modulating the activity of miRNAs by functioning as miRNA sponges [168]. Additionally, these molecules were found to be implicated in regulating the transcription and translation of specific target genes by binding to proteins [169]. circRNA-RNF111 was reported to positively contribute to the differentiation of preadipocytes into mature adipocytes. Data obtained from this study showed that circRNA-RNF111 masked the inhibitory activity of miR-27a-3p by functioning as an miRNA sponge and hence promoted adipogenic differentiation by increasing PPARγ expression [170]. circRNA-SAMD4A was found to regulate adipogenic differentiation by targeting and interacting with miR-138-5p within a circSAMD4A/miR-138-5p/enhancer of zeste homolog 2 (EZH2) feedback loop [171]. circRNA-H19 was reported to negatively regulate the differentiation of hADSCs. In an in vitro study conducted by Zhu et al., knockdown of hsa-circRNA-H19 expression significantly increased the expression of adipogenic-related genes (C/EBPα, PPARγ, sterol regulatory element-binding protein-1c (SREBP-1c), FABP4, ACC-1, lipoprotein lipase (LPl), and FAS) and promoted the accumulation of lipid droplets. circRNA pull-down and RNA immunoprecipitation (RIP) analysis demonstrated that circRNA-H19 targets and interacts with polypyrimidine tract-binding protein 1 (PTBP1), an RNA splicing factor previously identified to play a role in lipid metabolism-mediated pathways [172], and is implicated in the translocation of SREBP1 from the cytoplasm to the nucleus [173]. circRNA-MEF2C(2,3) was identified as a key negative regulator of adipose development and differentiation. miR-383 and miR-671-3p were shown to interact with circRNA-MEF2C(2,3) and form a complex, which binds to the 3′UTR region of MEF2C(2,3) target gene, hence modulating proliferation and adipogenic differentiation [174]. circRNA-HOMER1 was reported to suppress adipogenic differentiation. Overexpression of circRNA-HOMER1 significantly decreased the expression of adipose-specific marker genes (PPARγ, C/EBPα, C/EBPβ, and FABP4) and impaired the synthesis and deposition of fat droplets within the cytoplasm. Moreover, circRNA-HOMER1 was found to target miR-23b and SIRT1 activity [175]. Regarding BAT differentiation, circZEB1 was reported to promote brown adipogenic differentiation and thermogenesis. Overexpression of circRNA-ZEB1 determined elevated mRNA and protein expression levels of BAT-related marker genes, including UCP1, elongation of very long chain fatty acids protein 3 (ELOVL3), and PGC1α. Also, the expression of mitochondrial-specific genes (ATP6, carnitine palmitoyltransferase 1a (CPT1a), CPT2, and COX1) was observed to be increased. Data obtained from RIP and luciferase reporter analysis indicated that circRNA-ZEB1 can reverse the inhibitory function of miR-326-3p on lipid droplet accumulation in brown adipocytes by directly targeting its activity [176]. circRNA-Nrxn2 was shown to enhance WAT browning by increasing the polarization of M2 macrophages. circRNA-Nrxn2 was also determined to impair the activity of miRNA-103 by functioning as an endogenous miRNA sponge and to increase the levels of FGF10, resulting in the browning of WAT [177]. Additional examples of circRNA molecules acting as either positive or negative regulators of WAT/BAT differentiation are mentioned in Table 5.

## 7. Future Perspectives of Non-Coding RNAs as Potential Therapeutic Targets

Recently, there has been an increasing interest in identifying and reporting novel ncRNA transcriptional profiles. Hundreds of ncRNA molecules have been determined to function in regulating stem cell differentiation into different cellular lineages, including the adipogenic lineage. Numerous studies proposed ncRNAs as potential biomarkers and therapeutic targets in many diseases. miR-1 was reported as a promising therapeutic candidate for the modulation of thermogenesis and metabolic homeostasis in human adipocytes [184]. miR-10a-3p was identified as a novel therapeutic target for the suppression of adipose inflammation and treatment of associated metabolic disorders [185]. According to the literature, miRNAs have been reported to contribute to the development of chronic diseases, including diabetes, chronic kidney disease (CKD), obesity, and vascular disorders [186,187]. Current investigations regarding the role of miRNAs during adipogenesis provide a potential use of these molecules as inhibitors or mimics to treat these diseases. For example, studies using mimics/antimiRs for miR-103/107 [188], miR-208a [189], miR-21 [190], and let-7 [191] determined impaired adipogenesis and favored glucose homeostasis. However, the potential of miRNAs in clinical practice is to be further identified. Although numerous miRNA molecules have been reported to regulate adipogenic stem cell differentiation and different metabolic processes, the selection of specific miRNAs for stem cell-based and miRNA-patient-targeted therapies remains challenging but, at the same time, promising. Another interesting application of ncRNAs is their use as potential candidates for tissue repair and regeneration [192]. In the domain of tissue engineering, research on the regulation, stability, and delivery of ncRNAs for the treatment of obesity-related diseases such as diabetes is rapidly developing. For instance, in the study conducted by Li et al., a novel silk fibroin scaffold combined with exosomes for controlled miRNA delivery was developed to treat diabetic wounds. miRNA-146a was encapsulated in engineered exosomes and released to accelerate wound closure and promote diabetic wound healing. Data from this study reported enhanced collagen deposition, improved neovascularization, and reduced inflammation in diabetic mice upon treatment [193]. Another study showed that miR-21 could promote diabetic foot ulcer treatment in mice when loaded in a graphene oxide (GO)-based wound dressing scaffold. miR-21-5p was reported to bind and suppress the expression of PTEN and lncRNA-PVT1, hence eliminating the influence of damaged skin [194]. Furthermore, engineered exosomes derived from miR-132-overexpressing ADSCs were developed for promoting cutaneous tissue reconstruction of diabetic wounds. Both in vitro and in vivo studies showed that miR-132-exo significantly promoted angiogenesis and enhanced diabetic wound healing via the regulation of the NF-κB signaling pathway [195].

## 8. Conclusions

Multiple regulatory factors are involved in the complex process of adipogenesis. Emerging studies highlight the function of ncRNA molecules in regulating the development and function of adipose tissue. In this review, we were, for the first time, able to systematically present a summary on the role of different ncRNAs, focusing on the function of miRNAs as key mediators of adipogenic initiation or suppression of differentiation into white and brown adipose tissue. The function of numerous molecules, as well as their specifically targeted gene markers and molecular pathways, was presented. Additionally, adipose tissue presents the main source of circulating miRNAs, participating in both endocrine and paracrine signaling. Accumulating evidence shows that miRNAs, lncRNAs, and circRNAs can significantly impact important physiological and functional characteristics of white and brown adipose tissue. Not only were these molecules identified as major regulators of differentiation, but they were also determined to play a critical role in regulating glucose uptake, controlling lipid metabolism, modulating metabolic changes, and maintaining energy homeostasis. Elucidating the mechanisms of function of miRNAs and their subsequent gene targets contributes to our knowledge about the ability of these molecules to regulate adipogenic differentiation into white and brown adipose tissue. This, in turn, would offer a new promise of using these molecules as biomarkers and may allow the development of novel therapeutic approaches, particularly for personalized treatments targeting obesity, as well as for advancements in tissue engineering-based therapies.

## Figures and Tables

**Table 1:** Key miRNA molecules regulating different stages of WAT differentiation. Abbreviations: RUNX2: runt-related transcription factor 2; BMP2: bone morphogenetic protein 2; HDAC6: histone deacetylase 6; RB2/p130: retinoblastoma- related gene; E2F3: E2f transcription factor 3; TBX3: T-box 3; HMGA2: high-mobility group AT-hook 2; KLF7: kruppel-like factor 7; PPARγ: peroxisome proliferator-activated receptor gamma; EID-1: EP300 interacting inhibitor of differentiation 1; TGFBR2: transforming growth factor beta 2; PTN: pleiotrophin; ERK5: extracellular signal-regulated kinase 5; WNT1: wingless-type MMTV integration site family, member 1; TCF712: transcription factor 7 like 2; GSK3β: glycogen synthase kinase 3 beta; SIRT-: silent information regulator sirtuin-.

Differentiation Stage	Key miRNA Regulators	Target Gene(s)	Outcome	Reference(s)
Cell fate determination	miR-30a/d, miR-204, miR-320	RUNX2	promote adipogenesis	[62,63,64]
	miR-17, miR-106a	BMP2	promote adipogenesis	[65]
	miR-194	HDAC6	inhibits adipogenesis	[66]
	miR-637	Osterix	promotes adipogenesis	[67]
Clonal expansion	miR-17-92 cluster	RB2/p130	promotes adipogenesis	[68]
	miR-363, miR-93	E2F3, TBX3	inhibit adipogenesis	[69,70]
	let-7	HMGA2	inhibits adipogenesis	[71]
	miR-33b	HMGA2	inhibits adipogenesis	[72]
	miR-146b	KLF7	promotes adipogenesis	[73]
Terminal differentiation	miR-130	PPARγ	inhibits adipogenesis	[74,75]
	hsa-miR-138	EID-1	inhibits adipogenesis	[76]
	miR-21	TGFBR2	promotes adipogenesis	[77]
	miR-143	PTN, ERK5	promotes adipogenesis	[78]
	miR-148a	WNT1	promotes adipogenesis	[78,79]
	miR-210	TCF712	promotes adipogenesis	[80]
	miR-344, miR-709	GSK3β	inhibit adipogenesis	[81,82]
	miR-146b	SIRT1	promotes adipogenesis	[83]
	miR-93	SIRT7	inhibits adipogenesis	[70]

**Table 3:** Additional key miRNA molecules with their specific target gene(s) and their role in BAT differentiation, development, and function. Abbreviations: ADAM17: a disintegrin and metalloproteinase; UCP1: uncoupling protein 1; PPARγ: peroxisome proliferator-activated receptor gamma; FABP-: fatty acid binding protein-; ADRB1: beta-1 adrenoreceptor beta 1; PRDM16: positive regulatory domain zinc finger protein 16; IGF2: insulin-like growth factor 2; EGR1: early growth response protein 1; ATG7: autophagy-related 7; BAT: brown adipose tissue; C/EBP-: CCAAT-enhancer-binding protein-; BACE 1: beta-site amyloid precursor protein cleaving enzyme 1; cAMP: cyclic adenosine monophosphate; PDE1b: phosphodiesterase 1b; HIF1an: hypoxia-inducible factor 1-subunit alpha inhibitor; RUNX1t1: runt-related transcription factor 1; NDN: neurally differentiated embryonal carcinoma-derived protein; PGC1α: PPARγ coactivator 1 alpha; CD137: cluster of differentiation 137.

	miRNA	Function(s)	Target Gene(s)	Reference(s)
Positive regulators of brown adipogenesis	miR-26	impairs the browning process upon its repression regulates the expression of metalloproteinase ADAM17	UCP1, PPARγ, FABP3, ADRB1, PRDM16	[109]
	miR-129	regulates thermogenesis and energy expenditure represents a potential obesity biomarker	IGF2, EGR1, ATG7	[110,111]
	miR-328	favors BAT differentiation and increases C/EBPβ, UCP1 levels, and oxygen consumption impairs muscle progenitor commitment by regulating the switch between myogenic and brown adipogenic lineages	BACE1	[112]
	miR-378	regulates cAMP turnover in BAT and enhances brown adipocyte differentiation stimulates lipolysis	PDE1b	[113,114]
	miR-455	enhances thermogenic capacity in response to cold controls brown adipogenesis represents a potential therapeutic target for human metabolic disorders	HIF1an, Cidea, RUNX1t1, NDN PPARγ, C/EBPα, and C/EBPδ, UCP1, PRDM16	[115]
Negative regulators of brown adipogenesis	miR-27b	increases expression of specific BAT markers (such as UCP1, PRMD16, PGC1α) upon its knockdown reduces energy expenditure and increases fat accumulation	PPARδ, prohibitin, PRDM16, UCP1	[116,117]
	miR-34a	reduces adiposity, improves serum levels, and increases oxidative function upon its blockage	CD137, UCP1	[118]
	miR-106b-93 cluster	plays a role in energy homeostasis increased expression in obesity	UCP1, PRDM16, Cidea, PPARα, PPARγ, PGC1α, FABP4, adiponectin	[70]
	miR-133	inhibits BAT differentiation	PRDM16	[119,120]

**Table 2:** Additional key miRNA molecules with their specific target gene(s) and their role in WAT differentiation, development, and function. Abbreviations: WAT: white adipose tissue;PPARγ: peroxisome proliferator-activated receptor gamma; C/EBPα: CCAAT-enhancer-binding protein alpha; FABP4: fatty acid binding protein 4; CREB: cAMP-response element-binding protein; PTEN: phosphatase and tensin homolog; WNT1: wingless-1; TNFα: tumor necrosis factor-alpha; AP2: fatty acid-binding protein; ERK1/2: extracellular signal-regulated kinases 1/2; FGF21: fibroblast growth factor 21; LIFR: leukemia inhibitory factor receptor; SREBP1: sterol regulatory element-binding protein 1; LPL: lipoprotein lipase; CD36: cluster of differentiation 36; EID-1: early region 1-A-like inhibitor of differentiation 1.

	miRNA	Function(s)	Target Gene(s)	Reference(s)
Positive regulators of white adipogenesis	miR-148a-3p	promotes WAT differentiation upregulates mRNA and protein levels of PPARγ, C/EBPα, and FABP4 enhances intracellular triglyceride content contains a functional CREB domain	PTEN, WNT1	[79,92]
	miR-181a	promotes adipocyte differentiation accelerates the accumulation of lipid droplets and the synthesis of triglycerides represses TNFα function (a cytokine involved in regulating lipogenesis)	TNFα	[85]
	miR-375	enhances adipogenic differentiation increases mRNA levels of C/EBPα, PPARγ2 induces the accumulation of Ap2 and triglyceride suppresses ERK1/2 phosphorylation levels	ERK1/2	[84]
	miR-26b-5p	promotes adipocyte differentiation increases levels of lipid deposition and adipogenic-related marker genes dowregulates significantly FGF21 mRNA expression	FGF21	[93]
Negative regulators of white adipogenesis	miR-377-3p	decreases adipogenic differentiation downregulates expression of key-adipogenic markers (such as PPARγ, C/EBPα, and AP2) reduces intracellular lipid droplet accumulation	LIFR	[94]
	miR-182	impairs WAT differentiation suppresses the synthesis of lipid droplets inhibits the expression of adipogenic-related markers (such as C/EBPβ, PPARγ, adiponectin, and SREBP1) represses glucocorticoid-induced expression of C/EBPα	C/EBPα	[95]
	miR-27a	inhibits adipocyte differentiation by reducing the expression of key adipogenic regulator PPARγ decreases lipid accumulation represses mRNA expression levels of AP2, LPL, CD36, and adiponectin	PPARγ	[96]
	hsa-miR-138	inhibits adipogenic differentiation reduces lipid droplet synthesis and accumulation decreases the expression of C/EBPα, PPARγ, LPL, adiponectin, and FABP4	EID-1	[76]

**Table 4:** Additional lncRNA molecules with their specific target gene(s) and their role in WAT and BAT differentiation, development, and function. Abbreviations: PPARγ: peroxisome proliferator-activated receptor gamma; FASN: fatty acid synthase; AKT: protein kinase B; FOXO1: forkhead box O1; IGF-1: insulin/insulin-like growth factor-1; MAPK: mitogen-activated protein kinase; JNK: c-Jun NH2-terminal kinase; ATGL: Adipose triglyceride lipase; H3K4me3: histone H3 lysine 4 trimethylation; AcH3: acetylcholinesterase; FABP4: fatty acid binding protein 4; WNT: wingless; Dnmt: maintenance DNA methyltransferase 1; C/EBP-: CCAAT-enhancer-binding protein-; MLL3/4: mixed lineage leukemia 3/4; H3K4me3: histone H3 lysine 4 trimethylation; H3K27me3: histone H3 lysine 27 trimethylation; PA1: Pax2 transactivation domain interaction protein (PTIP)-associated protein 1; CTCF: CCCTC-binding factor; HuR: Hu antigen R; SIRT1: silent information regulator sirtuin 1; TAZ: transcriptional coactivator with PDZ-binding motif; ABL: abelson; RUNX2: runt-related transcription factor 2; BAT: brown adipose tissue; PGC1α: PPARγ coactivator 1 alpha; CELF1: CUGBP Elav-Like Family Member 1; hnRNPU: heterogeneous nuclear ribonucleoprotein U; EBF2: early B cell factor 2; WAT: white adipose tissue; ETC: electron transport chain; p38MAP: p38 mitogen-activated protein.

	lncRNA	Function(s)	Target miRNA/Gene(s)	Reference
Positive regulators of white adipogenesis	lncRNA-Acart	regulates preadipocyte differentiation and proliferation decreases cellular apoptosis	PPARγ	[129]
	PU.1 AS lncRNA	promotes adipogenesis through the formation of a sense-antisense RNA duplex with PU.1 mRNA	PPARγ, FASN	[130]
	lncRNA-SRA	enhances adipogenic differentiation increases glucose uptake and phosphorylation of AKT and FOXO1 in response to insulin stimulates IGF-1 signaling and inhibits phosphorylation of MAPK and JNK during early differentiation stages	AKT, FOXO1	[131]
	lncRNA-lncIMF2	promotes proliferation and adipogenic differentiation acts as a molecular sponge for miR-217 regulates the expression of specific adipogenic marker genes	PPARγ, ATGl	[132]
	lncRNA-MIR31HG	favors adipocyte lineage commitment in vitro and in vivo regulates the expression of active histone markers H3K4me3 and AcH3 in the promoter region of FABP4 suppresses WNT/β-catenin pathway	FABP4	[133]
	lncRNA-slincRAD	promotes early adipogenesis by allowing the commitment of growth-arrested cells into the cell cycle through hormone induction guides epigenetic factors to mediate the methylation of cyclin-dependent kinase inhibitor p21 promoter	DNMT1	[134]
	lncRNA-ADINR	promotes adipogenic differentiation by modulating transcription of C/EBPα in cis and recruiting MLL3/4 histone methyltransferase complex increases H3K4me3 and decreases H3K27me3 histone modification in the C/EBPα locus	PA1	[135]
Negative regulators of white adipogenesis	lncRNA-H19	inhibits adipogenic commitment of cells through epigenetic modulation of histone deacetylases forms a complex with miR-675	CTCF	[36]
	adipoQ (adiponectin) AS lncRNA	inhibits white adipose tissue formation through its transfer from the nucleus to the cytoplasm forms a complex with adipoQ mRNA and suppresses its translation	adipoQ mRNA	[136]
	lncRNA-CAAlnc1	impairs adipogenesis blocks the binding of HuR to adipogenic transcription factor mRNAs and decreases the expression of these factors	HuR	[137]
	lncRNA-ADNCR	inhibits adipocyte differentiation functions as a competing endogenous RNA (ceRNA) for miR-204 promotes SIRT1 upregulation (gene implicated in inhibiting adipogenic gene expression by targeting PPARγ activity)	miR-204	[138]
	LncRNA-Bmncr	impairs white adipogenesis by serving as a scaffold to allow the interaction of TAZ and ABL facilitates TAZ-RUNX2/PPARγ transcriptional complex assembly	PPARγ	[139]
Positive regulator of brown adipogenesis	lncRNA-XIST	regulates brown adipocyte differentiation controls metabolic disorders by preventing high-fat diet-induced obesity	C/EBPα	[140]
	lncRNA-BATE1	promotes brown adipogenesis by binding to the heterogeneous ribonucleoprotein U plays a significant function in thermogenesis regulates the expression of a set of genes related to brown adipogenesis and mitochondrial biogenesis and function	PPARγ, C/EBPα, C/EBPβ	[141]
	lncRNA-BATE10	favors full brown fat differentiation and development plays a role in the browning of white fat downregulates respiratory electron transport at the genome level decreases significantly the expression of selective-BAT marker genes upon its knockdown competes with PGC1α mRNA for the binding of CELF1 during BAT differentiation	PPARγ, C/EBPα, FABP4, PGC1α	[142]
	lncRNA-Blnc1	promotes brown adipogenesis by stimulating thermogenic gene expression acts by forming a ribonucleoprotein complex with hnRNPU and EBF2	EBF2	[143]
	lncRNA-AK079912	promotes brown tissue adipogenesis and WAT browning upregulates the expression of genes implicated in thermogenesis regulates lipid accumulation, mitochondrial copy number, and levels of mitochondrial ETC	PPARγ	[144]
Negative regulator of brown adipogenesis	lncRNA-uc.417	impairs brown adipogenesis and attenuates the thermogenic program suppresses moderately p38MAPK signaling pathway decreases significantly the expression of BAT- and mitochondrial-related marker genes attenuates mitochondrial respiration rate	PPARγ2, C/EBPβ	[145]

**Table 5:** Additional circRNA molecules with their specific target gene(s) and their role in WAT and BAT differentiation, development, and function. Abbreviations: WNT5B: wingless family member 5B; PPARγ: peroxisome proliferator-activated receptor gamma; C/EBP-: CCAAT-enhancer-binding protein-; PGC1β: PPARγ coactivator 1 beta; MEOX2: mesenchyme homeobox 2; BAT: brown adipose tissue; ATGL: Adipose Triglyceride Lipase; RXRA: Retinoid X Receptor Alpha; ATGL: adipose triglyceride lipase.

	circRNA	Function(s)	Target miRNA/Gene(s)	Reference
Positive regulators of white adipogenesis	circRNA-CDR1as	regulates positively adipogenic differentiation acts via the miRNA-7-5p-/WNT5B pathway	miRNA-7-5p, WNT5b	[178]
	circRNA-ATXN2	promotes adipogenic differentiation increases the expression of PPARγ and C/EBPα favors lipid droplet synthesis and accumulation inhibits proliferation and promotes apoptosis	PPARγ, C/EBPα	[179]
Negative regulators of white adipogenesis	circRNA-FUT10	inhibits adipocyte differentiation by sponging miRNA-let-7 promotes adipocyte proliferation	let-7c/let-e, PGC1β	[180]
	circRNA-INSR	impairs adipogenic differentiation acts via miR-152/MEOX2 pathway	miR-152	[181]
Positive regulator of brown adipogenesis	circRNA-0001017	regulates brown adipogenesis by interacting with miR-503	miR-503	[182]
Negative regulator of brown adipogenesis	circRNA-Ogdh	impairs BAT differentiation promotes lipolysis by upregulating the expression of ATGL (key lipolysis protein) suppresses lipid droplet accumulation downregulates the expression of C/EBPα, C/EBPβ, PPARγ, and RXRA	miR-34a-5p, ATGL	[183]

## Data Availability

No new data were created with this review.
